# Patients’ Views on AI for Risk Prediction in Shared Decision-Making for Knee Replacement Surgery: Qualitative Interview Study

**DOI:** 10.2196/43632

**Published:** 2023-09-18

**Authors:** Daniel J Gould, Michelle M Dowsey, Marion Glanville-Hearst, Tim Spelman, James A Bailey, Peter F M Choong, Samantha Bunzli

**Affiliations:** 1 St Vincent's Hospital Department of Surgery University of Melbourne Melbourne Australia; 2 Department of Orthopaedics, St Vincent's Hospital Melbourne Melbourne Australia; 3 School of Computing and Information Systems, University of Melbourne Melbourne Australia; 4 School of Health Sciences and Social Work, Griffith University Brisbane Australia

**Keywords:** artificial intelligence, qualitative research, semistructured interviews, knee replacement, risk prediction, patient perception, patient understanding, patient preference, patient perspective

## Abstract

**Background:**

The use of artificial intelligence (AI) in decision-making around knee replacement surgery is increasing, and this technology holds promise to improve the prediction of patient outcomes. Ambiguity surrounds the definition of AI, and there are mixed views on its application in clinical settings.

**Objective:**

In this study, we aimed to explore the understanding and attitudes of patients who underwent knee replacement surgery regarding AI in the context of risk prediction for shared clinical decision-making.

**Methods:**

This qualitative study involved patients who underwent knee replacement surgery at a tertiary referral center for joint replacement surgery. The participants were selected based on their age and sex. Semistructured interviews explored the participants’ understanding of AI and their opinions on its use in shared clinical decision-making. Data collection and reflexive thematic analyses were conducted concurrently. Recruitment continued until thematic saturation was achieved.

**Results:**

Thematic saturation was achieved with 19 interviews and confirmed with 1 additional interview, resulting in 20 participants being interviewed (female participants: n=11, 55%; male participants: n=9, 45%; median age: 66 years). A total of 11 (55%) participants had a substantial postoperative complication. Three themes captured the participants’ understanding of AI and their perceptions of its use in shared clinical decision-making. The theme *Expectations* captured the participants’ views of themselves as individuals with the right to self-determination as they sought therapeutic solutions tailored to their circumstances, needs, and desires, including whether to use AI at all. The theme *Empowerment* highlighted the potential of AI to enable patients to develop realistic expectations and equip them with personalized risk information to discuss in shared decision-making conversations with the surgeon. The theme *Partnership* captured the importance of symbiosis between AI and clinicians because AI has varied levels of interpretability and understanding of human emotions and empathy.

**Conclusions:**

Patients who underwent knee replacement surgery in this study had varied levels of familiarity with AI and diverse conceptualizations of its definitions and capabilities. Educating patients about AI through nontechnical explanations and illustrative scenarios could help inform their decision to use it for risk prediction in the shared decision-making process with their surgeon. These findings could be used in the process of developing a questionnaire to ascertain the views of patients undergoing knee replacement surgery on the acceptability of AI in shared clinical decision-making. Future work could investigate the accuracy of this patient group’s understanding of AI, beyond their familiarity with it, and how this influences their acceptance of its use. Surgeons may play a key role in finding a place for AI in the clinical setting as the uptake of this technology in health care continues to grow.

## Introduction

### Background

With the growing prevalence of advanced knee osteoarthritis, more people will be faced with the decision to undergo arthroplasty surgery [1]. This is a complex decision that requires patients to consider the benefits and risks of surgery. Trust in the surgeon is critical as patients and surgeons engage in a decision-making process in which patients align their expectations with what can realistically be achieved with knee replacement surgery [2]. Clinical decision aids promote shared decision-making between patients and clinicians by improving patient knowledge and involvement [3]. The definition of *shared decision-making* used in this study is derived from prior literature [4]: “an approach where clinicians and patients share the best available evidence when faced with the task of making decisions, and where patients are supported to consider options, to achieve informed preferences.” Even relatively complex information from decision aids is comprehensible, provided the information is presented in a digestible and user-friendly manner [5]. Knee replacement surgeons have demonstrated openness to using decision aids to enhance communication and informed consent [6].

Decision aids are increasingly using machine learning (ML) to process large volumes of complex data [7], which could facilitate personalized prognostication. ML is a branch of artificial intelligence (AI), and although these technical terms are distinct, they are often conflated, which causes confusion around their meaning [8,9]. In this study, the following working definition of *machine learning* was used: “a subset of AI in which algorithms are trained on data sets to become machine learning models capable of performing specific tasks” [10]. This is distinct from “AI,” which is defined as “computer software that mimics human cognitive abilities in order to perform complex tasks that historically could only be done by humans, such as decision-making, data analysis, and language translation” [10]. However, as previously mentioned, the terms are often conflated, which results in many relatively simple ML decision aids being labeled as “AI” when this may not be the case according to the strict definitions. In light of these terminological nuances and lack of consistency in the way these terms are used, it is important to explore patients’ views on AI in shared decision-making, even when most decision aids do not use “AI” in the strict technical sense.

### Prior Work

AI is gaining prominence in orthopedics [11,12]. Its application in predicting the risk of postoperative outcomes is growing in popularity for patients undergoing knee replacement surgery [13,14], and AI decision aids are increasingly used to support decision-making [14,15]. However, the “black box” nature of some AI algorithms is a potential concern for both clinicians [16] and patients [17]. A black box is an uninterpretable model [18] built with advanced algorithms using many predictors that may interact with one another in complex ways that are clinically meaningless [19]. Contrasting this are interpretable risk scores using predictors with intuitive clinical relevance, such as the Fracture Risk Assessment Tool for predicting fractures in patients with osteoporosis [20]. This is an important distinction between risk estimates from AI decision aids compared with those from non-AI decision aids, because AI has the potential to perpetuate or even exacerbate inequities when decisions are made on the basis of black box predictions [21]. A recent large survey study of the Australian public indicated a lack of support for unexplainable hospital algorithms [17].

A recent review found that patients from diverse clinical populations and geographic regions believed that AI could provide a second opinion to clinicians and improve access to care by providing patients with remotely available personalized information at any time at minimal cost [22]. However, concerns included a lack of clinician oversight and inability to fully share in decision-making. Most of the studies included in this review were surveys; few studies used qualitative interviews to explore patients’ attitudes, values, and preferences toward AI. In health literature, a single qualitative study [23] used cognitive interviews to develop a questionnaire to measure the acceptance of AI among patients scheduled for computed tomography scan, magnetic resonance imaging, or conventional radiography. Incorporating patient voices into the development and implementation of decision aids using ML is important to ensure that they address patient concerns and are of maximum possible benefit [24,25]. However, the views of patients undergoing knee replacement surgery on the use of AI for risk prediction in shared decision-making have not been explored.

### Goals of This Study

The aim of this study was to explore the understanding and attitudes of patients who underwent knee replacement surgery regarding AI use in the context of risk prediction for shared clinical decision-making. Patients might not be aware of the types of decision aids that were used in shared decision-making; therefore, this was seen as an opportunity to bring it to the attention of people who had undergone knee replacement surgery. This enabled their views to be ascertained to better understand their perspective on the growing recognition of the importance of the right of health care recipients to be more informed about and involved in decisions relating to their treatment [26,27]. The aim of this study was not to provide a representative view of all patients who underwent knee replacement surgery. Rather, the goal was to harness the power of qualitative research techniques to gain a deeper understanding from a variety of diverse viewpoints. The findings of this qualitative exploration will inform the implementation of AI decision aids in clinical practice and lay the foundation for future research into the acceptance and uptake of these tools by patients undergoing knee replacement surgery.

## Methods

### Ethical Considerations

Ethics approval was granted by the Human Research Ethics Committee of St. Vincent’s Hospital, Melbourne, Australia (reference: Low-Risk Research 117/21).

### Design

**Figure 1:**
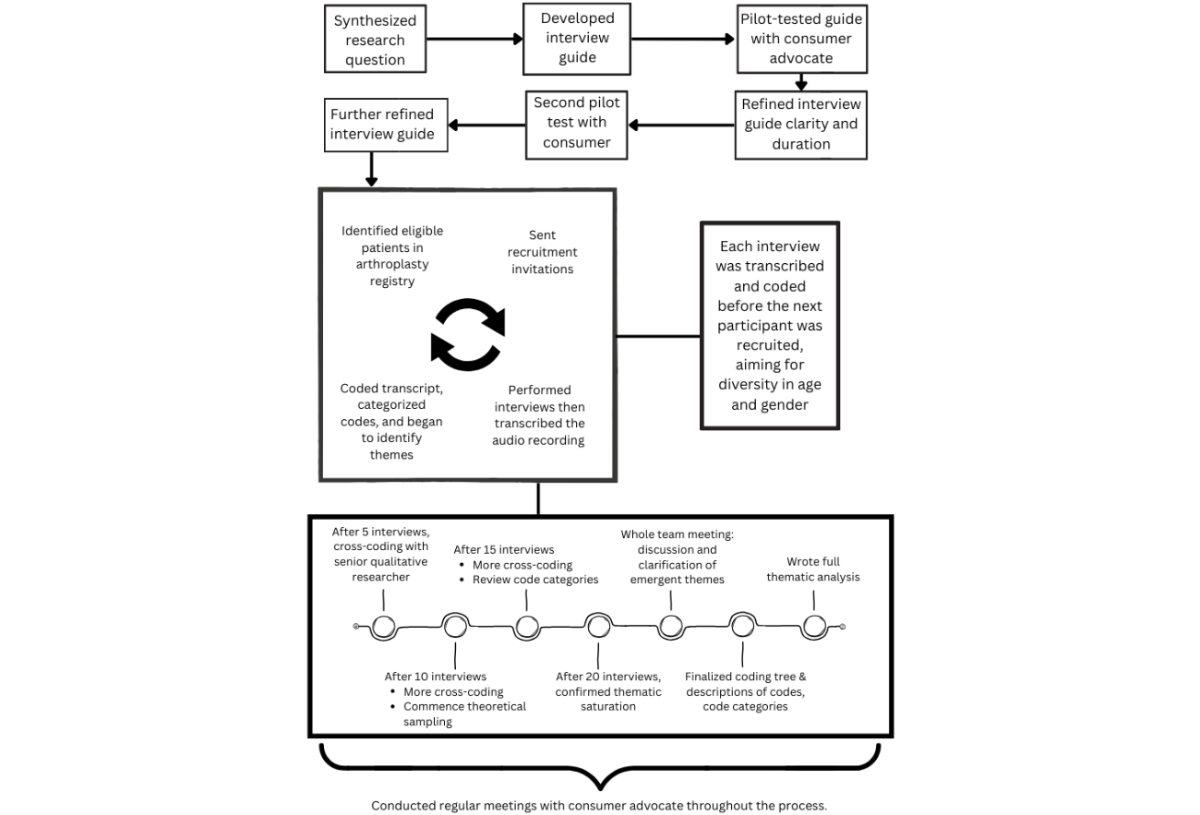
Study overview.

In order to orientate the reader before expanding upon the methodological details of each aspect of the study, Figure 1 provides an overview of the study.

Understanding of AI by patients who went knee replacement surgery and their views on its application in shared clinical decision-making were explored through semistructured qualitative interviews and analyzed using reflexive thematic analysis [28]. This enabled the research team to interpret participant responses through their lens as a team comprising a doctor of medicine (MD)–doctor of philosophy (PhD) candidate (author DJG) working with AI for patients undergoing knee replacement surgery, a qualitative researcher and physiotherapist (author SB), an epidemiologist and orthopedic nurse with experience in predictive tool development (author MMD), an academic orthopedic surgeon (author PFMC), a biostatistician (author TS), an academic AI expert (author JAB), and a consumer with personal experience in total joint replacement surgery (author MG-H).

This study followed the COREQ (Consolidated Criteria for Reporting Qualitative Research) guidelines [29] (Multimedia Appendix 1 [29]).

### Recruitment

Recruitment took place between August and December 2021. Patients who had undergone knee replacement surgery were identified from the joint replacement registry of a single tertiary hospital in Australia, with a large geographically, socioeconomically, and culturally diverse referral base. This registry has been described previously [30]. Purposive sampling [31] was used to identify eligible patients based on age and sex to obtain a broad range of perspectives from a diverse sample. Theoretical sampling [32] was used to challenge emerging themes through the recruitment of patients who had experienced a postoperative complication to ensure that a broad range of perspectives were represented in the final sample. This final criterion was selected because the value of AI for risk prediction was expected to potentially differ among those who had experienced a postoperative complication, given that they had experienced one of the outcomes that one might try to predict using AI. Theoretical sampling is an important component of robust qualitative research [33], in line with the underpinning constructivist epistemology, whereby researchers seek to construct the meaning by testing emerging interpretations of the data through the recruitment of participants who can provide diverse perspectives.

Patients were sent a letter or email briefly introducing the study and explaining that the interviewer and study coordinator was a male MD-PhD candidate (DJG) who, as part of his PhD research, would investigate the development of AI decision aids for patients undergoing knee replacement surgery. Patients were contacted via telephone 1 week later by the study coordinator for recruitment. Verbal consent was obtained before the commencement of each interview. None of the patients had any preexisting relationships with the members of the research team.

The sample size was determined by thematic saturation [34]. Data were analyzed simultaneously with data collection. This concurrent analysis was conducted until it was determined that no new concepts were identified in subsequent interviews, at which point recruitment was ceased.

### Data Collection

Data were collected through semistructured telephone interviews carried out by the study coordinator (DJG), a male MD-PhD candidate having experience in patient interviews through medical school education, and further training in qualitative interviews through qualitative methodology workshops was facilitated by the experienced senior qualitative researcher (SB). The interview guide was cocreated and pilot-tested with the consumer coauthor (MG-H). The guide necessarily evolved reflexively throughout the concurrent processes of interview and analysis. This is an important component of robust qualitative research because the first version of the interview guide is the researchers’ best attempt to capture data that are relevant to the research question, but it is impossible to know what will be found until data collection begins [35]. Changes were made following discussions between the study coordinator (DJG), the consumer advocate (MG-H), and the senior qualitative researcher (SB) to clarify, define, and challenge emerging interpretations. The final version of the interview guide was provided in Multimedia Appendix 2. The interview guide consisted of open-ended questions designed to establish the participants’ experience of shared clinical decision-making for their surgery, and subsequently, the participants explored their understanding of AI and views on the use of AI in the shared clinical decision-making process. The interview was carefully structured to ask participants to explain their own understanding of AI before a functional, nontechnical definition was provided. This was followed by the example of Deep Blue, the chess algorithm which was selected in consultation with the consumer advocate (MG-H) as an accessible, recognizable example of AI [36]. DJG checked that participants understood the concept before providing them with a hypothetical clinical example of AI on which the remaining questions were structured. Participants were able to be accompanied by a family member or support person during the interviews to ensure they were comfortable participating and to assist in the interpretation of some terminology and questions for participants whose first language was not English. Field notes were not taken during the interviews, but a summary of the interviews was recorded by DJG immediately following each interview. Repeat interviews were not carried out.

Interviews were audio recorded, transcribed, and uploaded into qualitative data management software (NVivo data management package [version 12.0; QSR International]) to facilitate the analysis.

### Data Analysis

The constant comparative technique was used throughout the analysis process, in which the researchers cycled back and forth between emerging themes and transcripts to ensure that interpretations remained grounded in the participants’ perspectives [37,38].

The study coordinator (DJG) identified the initial codes in each transcript through an inductive coding process shortly after each interview was completed. The study coordinator regularly met with the senior qualitative researcher (SB) to develop a coding framework. This process took place iteratively to expand, collapse, or alter codes as new transcripts were generated and earlier transcripts were recoded [39]. A third researcher (MMD) was available to be consulted whenever there were differences of opinion on the interpretation of data or codes. Once the research question had been comprehensively addressed and no further changes to the codes, or code categories, were deemed necessary, the study coordinator (DJG) applied the final coding framework to all transcripts. A subset of transcripts was coded by the second author (SB) to ensure that the final coding framework captured all the relevant data.

Codes were categorized by the study coordinator (DJG) through discussion with a second researcher (SB) to identify similarities, differences, and relationships between codes. This process also made the data more amenable to the abstraction of themes that were pertinent to the research aim. Findings that did not fit the pattern emerging from the data were given due consideration, but the research aim was ultimately used to guide the process by which findings relevant to the patterns were retained, and others were omitted from the final thematic analysis. For example, participant 15 (a 72-year-old man) worked in the IT sector for many years and had a strong personal interest in AI. There were many interesting points of discussion that were raised throughout the interview based on this participant’s professional experience and personal reading, but only those that were relevant to the research question were retained in the final thematic analysis.

Themes were generated using reflexive thematic analysis [28] based on the patterns identified in the data, with reference to the research aim. These themes, along with illustrative quotes, were discussed by the wider research team (DJG, MMD, TS, JAB, MG-H, PFMC, and SB). Information pertaining directly to the research aims was differentiated from the information that provided context to the participants’ views. This contextual information was not incorporated into the themes but helped inform their development by providing a better understanding of the participants’ circumstances and state of mind when considering the research questions.

## Results

### Recruitment

A total of 42 patients were approached; of them, 20 (48%) participants consented to participate in the study. Of the 22 (N=42, 52%) participants who did not participate, 14 (64%) did not respond, and the remaining 8 (36%) declined for the following reasons: 3 were not interested, 1 cited “research fatigue,” 1 stated that it was too much to take on considering a recent serious diagnosis, 1 did not feel up to it, 1 was not prepared to participate, and 1 did not have time. Each time a patient declined, a patient with similar demographic characteristics was sought based on age and sex and whether the patient had experienced a postoperative complication. The mean interview duration was 45 (SD 15) minutes.

Of the 20 participants, 11 (55%) were female patients. The median age of the participants was 66 (IQR 49-81) years. A total of 11 (55%) patients experienced a short-term postoperative complication that resulted in a deviation from routine care, such as additional unplanned procedures, delay in discharge, or readmission. Although 19 (95%) participants received total knee replacement, 1 (5%) participant (participant 3) underwent bilateral unicompartmental knee replacements.

### Coding Tree

**Figure 2:**
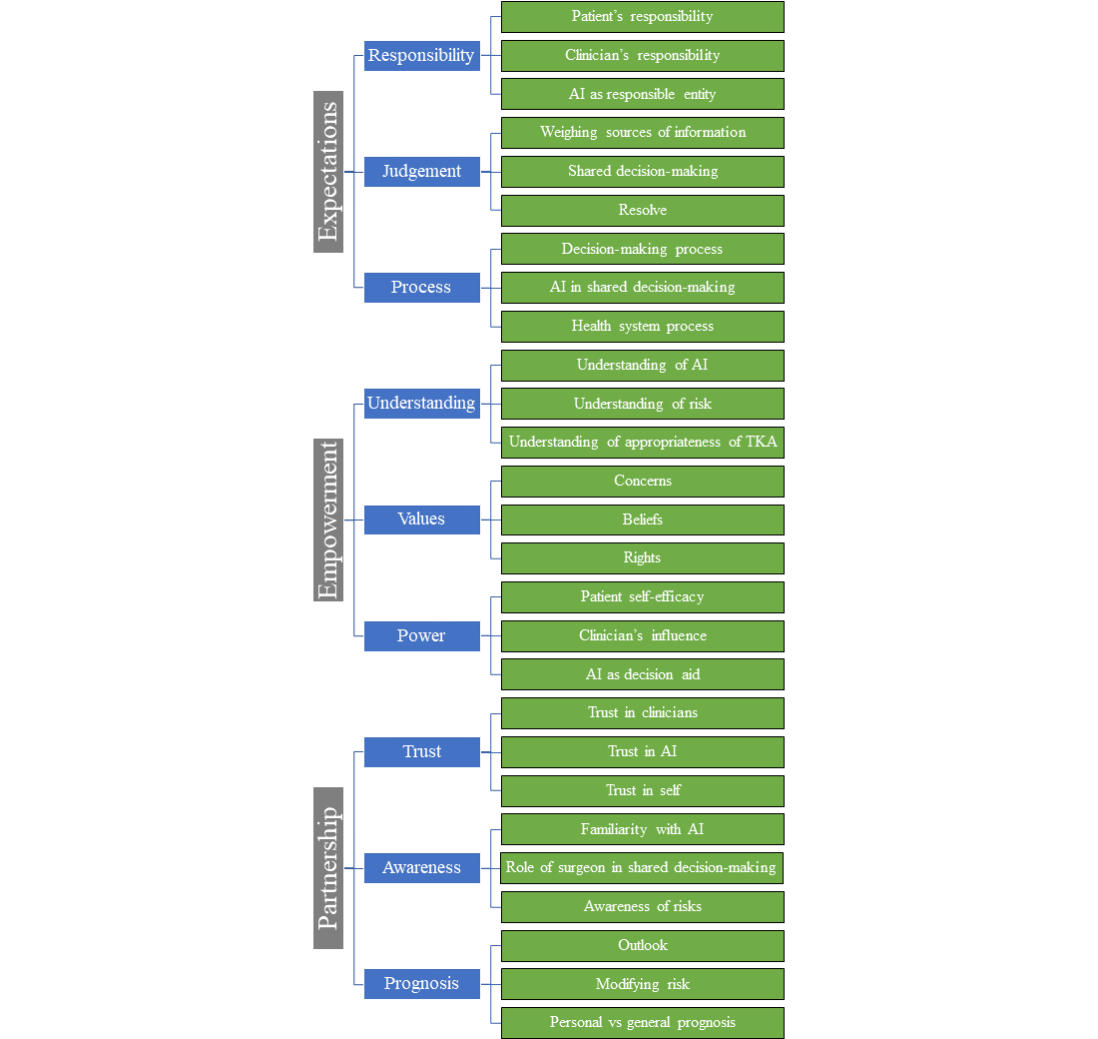
Themes, code categories, and codes. AI: artificial intelligence; TKA: total knee arthroplasty.

All instances in which the 2 coders (DJG and SB) had a difference in opinion regarding the interpretation of data were resolved through discussion, without the need to consult the third researcher (MMD). The iterative process of coding resulted in a coding tree comprising 27 codes grouped into 9 categories, with 3 codes per category. The final coding framework is illustrated in Figure 2.

For readers interested in better understanding the coding tree, detailed descriptions of each code and code category are provided in Multimedia Appendices 3 and 4, respectively. The emergent themes are described in detail in the *Thematic* *Analysis* section*.*

### Thematic Analysis

#### Overview

In total, 3 broad themes captured the participants’ understanding of AI and their perceptions of its use for risk prediction in shared clinical decision-making: Expectations, Empowerment, and Partnerships. Each theme is described in narrative form in the following sections, supported by quotes selected from a range of participants. Quotes are provided along with the participants’ age (years) and sex (female or male) in parentheses.

#### Theme 1: Expectations—“I think I need surgery”

This theme describes how participants used their judgment to make decisions based on their expectations of themselves, their clinicians, and the sources of information they encountered. Participants perceived that it was their responsibility to advocate for their own needs based on their level of pain, function, and quality of life. Clinicians were seen as being responsible for offering surgery to suitable candidates and performing them safely. AI could be viewed as another source of information, or as a responsible entity with decision-making capability. In either case, it could inform but not dictate their decision-making process.

Having navigated their way through primary care, the participants reported that by the time they reached the surgical consultation, they perceived that joint replacement surgery was their only option and were no longer considering nonsurgical treatment options. For most, this position was supported by the surgeon:

[W]hen I came up to see the surgeon, he said it was really bad and if I didn’t have it done, that I wouldn’t be able to carry on doing what I wanted to do

Participant 8, male, aged 71 years

Some participants believed that it was their responsibility to convince the surgeon of their need for surgery and perceived that a decision aid could play a role in helping patients convince surgeons. For example, participant 17 (male, aged 49 years) suggested that AI could provide more personalized information about the patient’s likely outcome such that the surgeon and patient could be better prepared:

I think it’s a great reference tool for them, but possibly not be in the position to be able to dictate terms.

Participant 17, male, aged 49 years

However, concerns were raised regarding the ability of a decision aid to provide individualized prognostic information, because accurately accounting for differences between people is very difficult:

[W]e all are built the same, but that doesn’t mean physically the same.

Participant 3, male, aged 53 years

Indeed, one overarching expectation among the participants was that AI would not be used to deny them the treatment they needed. Participants recognized that there was no right or wrong approach to decision-making for surgery, provided that the patient’s right to choose was respected. In recognition of the potential for AI to inform patient decision-making, one participant suggested that the AI tool could be administered to the patient before their consultation:

I’d like to see it shipped out to the patient to answer a whole bunch of questions.... So that when you turned up at the surgeons, you already had all that stuff.

Participant 15, male, 72 years

When asked to consider a hypothetical situation about whether the patient or the clinician should have the final say on whether to use AI when the clinician did not wish to use it, but the patient did, participants’ opinions were evenly divided. These 2 groups did not differ based on age, sex, preexisting familiarity with AI, or postoperative complication status. Contrasting views were characterized by a high degree of trust in the clinician on the one hand and a greater emphasis on patient autonomy on the other:

I think the surgeon should have the final say...because you’re in their hands

Participant 11, male, aged 80 years

I would not be using that surgeon and I wouldn’t let that surgeon near me

Participant 14, female, aged 73 years

There are likely many complex reasons why patients may hold these views on an individual level, but helping patients achieve a better understanding of AI may increase their affinity for it [8]. An unanticipated finding of a prior study involving members of the public suggested that the use of explanatory scenarios had an educational effect, whereby participants developed a more realistic view of the capabilities of health AI [40].

**Figure 3:**
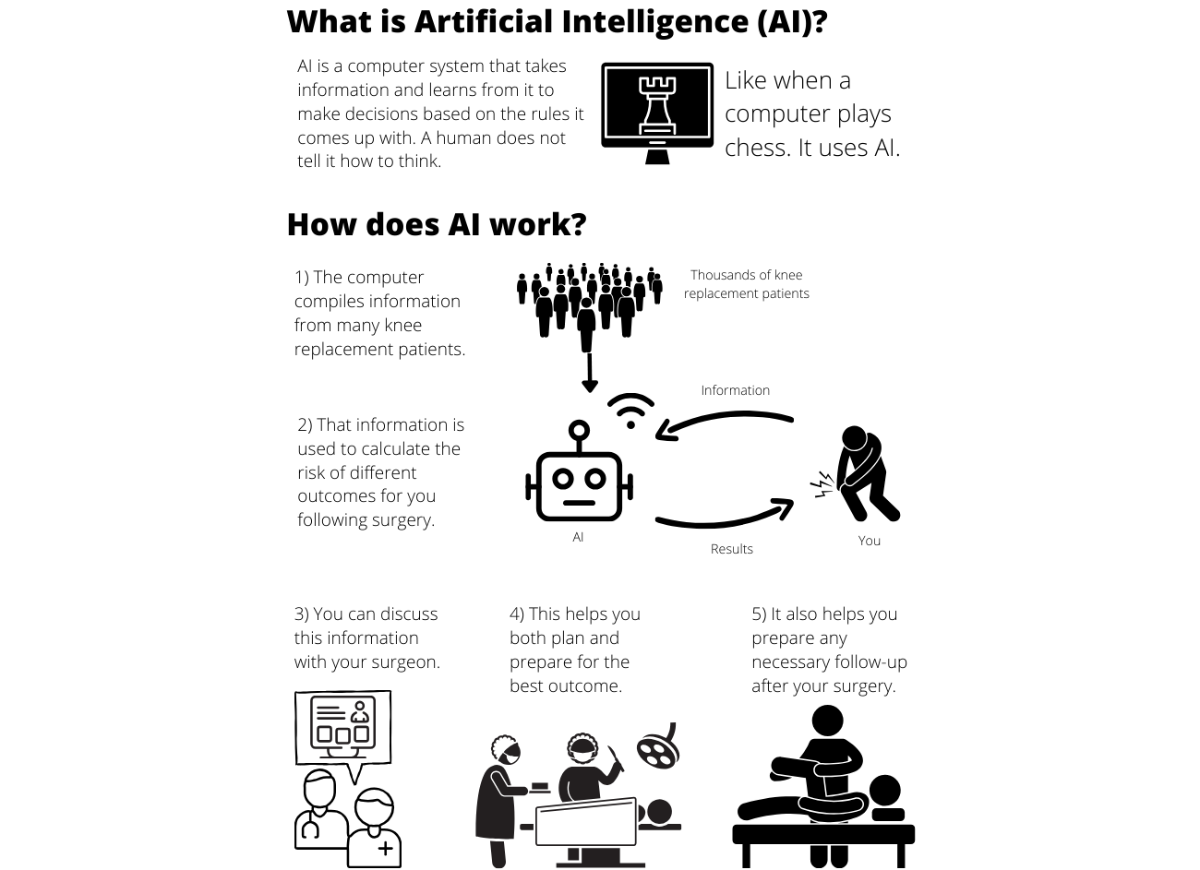
Example of educational flyer for patients.

Given the limited time available in a consultation for surgeons to explain such concepts to patients, educational material provided to patients before the consultation could also be an effective method of improving the understanding of the way AI enhances risk prediction for shared clinical decision-making. This could inform the patient’s decision regarding whether they want their data to be used for personalized risk prediction in the consultation and help them understand the benefit of allowing their data to be used to further train and improve risk prediction tools. To this end, the infographic presented in Figure 3 was codeveloped with the consumer advocate (author MG-H) to give patients the opportunity to enhance their understanding of AI for risk prediction and, therefore, use the limited time available in the clinical consultation for more pointed questions pertaining to their personalized prediction and how this information can inform their decision to proceed with surgery.

#### Theme 2: Empowerment—“It’s all about informed consent”

In this theme, participants suggested that making a truly informed decision required a satisfactory understanding of their risk profile and likelihood of a positive outcome following surgery. Their values needed to be considered, and their power to make the final decision needed to be respected. AI could enhance their understanding, empowering them to act in accordance with their values and preferences.

Some participants perceived that the critical aspect of patient empowerment was informed consent and believed that AI could provide information to develop realistic expectations:

[I]t’s all about informed consent, and the more informed the patient is, the better the decision-making is about the consent.

Participant 15, male, aged 72 years

One participant likened the value of AI to enhance the understanding of the risk profile and likelihood of a positive outcome following surgery to the modern car. She explained that cars had sensors to detect things that humans could not and, if ignored, these things might cause problems down the road:

[T]he car will tell you...the tyre is a little bit low. I look at it with my eyes, it doesn’t show, but the car is telling me there is something.... If I ignore it, I’m going to have flat tyre.

Participant 19, female, aged 61 years

By detecting unique patient characteristics that are undetectable to humans, the participant suggested that AI could enhance the understanding of risk profiles. Enhanced understanding could, in turn, empower people to advocate for their own needs based on their personal values. One participant further suggested that AI could empower them with knowledge about how to manage risk and optimize their likelihood of a successful outcome from surgery:

I would ask the doctor how I could improve my situation based on all the information given by the computer and by the doctor, but then it depends on how much the doctor...thought of the AI’s advice.

Participant 1, female, aged 61 years

As would be expected among a clinical sample, some participants were risk averse, whereas others were willing to accept a higher risk for a relatively small benefit. When presented with a hypothetical scenario of an AI decision aid telling them that they could potentially benefit from surgery even if the risk of complication was relatively high (Multimedia Appendix 2), one participant responded as follows:

[I]f I go through life for the next 15 years in complete agony, I’d rather...take the risk on the artificial intelligence fixing me.

Participant 16, male, aged 67 years

A few participants voiced caution, however, suggesting that patients need to be fully informed of the potential consequences and liability concerns, should they choose to reject the AI’s advice:

I’m going to carry the consequences for it...you cannot bypass without good reason. If you just bypass it, you’re going to carry the mistake.

Participant 18, female, aged 61 years

#### Theme 3: Partnership—“Ultimately I trust the surgeon”

This theme describes how patients enter a partnership with their surgeons and how AI could function as a member of this partnership, providing patients with personalized information about their prognosis. Trust in the surgeon, in AI, and in self were crucial aspects of this partnership. Participants’ existing awareness of the risks of surgery, and their familiarity with AI decision aids, could interact with their perception of the role that surgeons play in shared decision-making. Participants’ outlook on their potential to benefit from surgery influenced the degree of risk that they were willing to accept. AI could inform strategies to mitigate modifiable risks.

The ultimate decision to proceed with surgery was viewed by the participants as a partnership between patients and surgeons. Some participants positioned AI as an independent thinking entity, akin to a second opinion. These participants recognized that AI enabled risk prediction to be made based on the experiences of many people fed into the computer, unlike a clinician who only had their clinical experience to draw on, thus enabling more sensitive, precise predictions:

Any changes that are happening...around the world...like, for example, COVID now, we don’t know what sort of impact it’s having on everyone’s health, and so they might pick up something that’s happening...we learn from each other and I think becoming aware of the environmental factors that are affecting the world rather than just a few people.

Participant 13, female, aged 51 years

Trust in the information fed into the computer and mechanisms for data protection was raised by one participant:

[H]acking...could someone be trying to be nasty?

Participant 6, female, aged 81 years

When given the choice between an interpretable AI with reasonable performance (AI 1) and an uninterpretable AI (ie, “black box,” AI 2) with excellent performance (Multimedia Appendix 2), participants were evenly divided. These 2 groups did not differ based on age, sex, familiarity with AI, or postoperative complication status. Preference for AI 1 accompanied a feeling that knowing how the AI thinks was important to identify how to improve it:

[I]t’s like when...kids get something wrong, it could be something earlier on, but no one picked up or forgot or so on.... And it could be simply fixed up like that

Participant 13, female, aged 51 years

Contrasting this was the view that AI 2 would be preferable because if it achieved the desired result, it was less important to know how it worked:

I use the laser cutter at the wood workshop. And I don’t know how that works. But I still use it because it’s functional.

Participant 4, female, aged 58 years

Most participants perceived that although AI could offer valuable information to inform decision-making, it was the clinician’s empathy and humanity that were crucial in the decision to proceed with surgery. Participants tended to have a high level of trust in their surgeons, believing that they would only offer surgery if they believed the benefits outweighed the risks. Some participants trusted that the risks had been accounted for and did not want to know the details of what could go wrong, often believing that surgeons and their clinical team were equipped to manage any potential adverse events. For example, one participant commented that although infection was an important possible complication after surgery, she did not believe it was a reason to withhold surgery:

...I’d discuss it with the doctor and say, well, look, you get an infection, you can have antibiotics.... But I don’t think I’d stop the operation worrying that I might get an infection.

Participant 6, female, aged 81 years

### Demographic Profile of Themes

In addition to providing demographic information (age and sex) for each illustrative quote included in the main *Thematic Analysis* section, further information on the demographic profile of each theme is provided in the following paragraphs.

Although the notion of using statistics to describe themes goes against the conventions in qualitative research [41], Multimedia Appendix 5 depicts the total number of substantive quotes identified across all 20 interview transcripts that were meaningfully related to each theme. These were initially counted at the code level and were then aggregated to the level of themes. The demographic profile of each theme is depicted in the table in terms of the number (and proportion) of quotes from each age group and sex. This is presented to demonstrate the relatively even spread of demographic characteristics for each theme, without any obvious major demographic differences. However, it is important to reiterate that this is a qualitative study, and as such, these numbers need to be interpreted in a very broad sense and not in terms of statistical precision or generalizability.

### Additional Questions Providing Further Context for Findings

Multimedia Appendix 6 contains information on questions that required a closed response. The responses were then expanded upon in the interview, but this table was intended to provide a brief snapshot of these responses.

The following section provides the context for the findings displayed in Multimedia Appendix 6. This was achieved through the description of the broad demographic features of the respondents and the use of relevant quotes. Demographic features were not highlighted for the purpose of pointing out statistical differences between participants who responded differently to these questions. Rather, they were provided to demonstrate the diversity of the participants who held different views. As such, the exact numbers and proportions are not presented here in the main text, and instead terms such as “most” or “few” were used [41].

Around half of the participants (11/20, 55%) indicated that they had some understanding of the term AI before being provided with a working definition and then discussing it in the context of shared clinical decision-making. Approximately half (6/11, 55%) of those with an understanding were female, which was similar to those who indicated no prior understanding. Each group comprised a range of age categories (<60, 60-70, and >70 years). Most (8/11, 73%) of those who reported some prior understanding of AI had experienced a substantial postoperative complication compared with a minority of those who had no prior understanding. Some participants understood AI to be something designed by humans to help them in making decisions; participant 15 (male, aged 72 years) characterized it as “the ability to organise slash arrange computing capacity and power to be able to assist humans to make better decisions,” and participant 13 (female, aged 51 years) characterized it as “using computers in a way that they can think and assist the person using it.... You put in a code or something or an algorithm or whatever, and they come back and assist you.” A few participants understood AI to be something designed by humans to replace humans in the completion of certain tasks:

I guess artificial intelligence means that machines are doing something that people used to do

Participant 14, female, aged 73 years

I understand artificial intelligence and I understand...about robotics. I’ve seen a lot of things on TV with robots doing operations and things like that and robots doing machine work in factories and making cars and things like that.

Participant 16, male, aged 67 years

One participant understood AI to be a self-aware, self-improving thing:

Yeah, so self-aware, literally able to make decisions based on a result, as opposed to a human who can do the same thing five times and not know they are doing it wrong. Literally, artificial intelligence is supposed to be able to diagnose the result of any one action and be able to change that action.

Participant 17, male, aged 49 years

A few participants understood AI to be a sort of mind-body intelligence whereby the mind is getting accustomed to an artificial thing, such as a knee prosthesis, being in the body. Participant 3 (male, aged 53 years) characterized it as “what it means to me is...replacing natural parts with foreign parts,” and participant 1 (female, aged 61 years) stated “Yeah, so how the artificial intelligence...interacts with the human body.” Participant 1 extended her definition with an analogy from her experience as a swim teacher, and she likened the process of her body adapting to the prosthesis to the process by which she developed a way of communicating with a deaf student she once taught:

I think it would be a process in time where your body would have to get used to that. I used to also have a student, who I taught swimming, and she was one of the first children to have a cochlear implant.... She couldn’t use that within the water...but we managed to develop our own form of language.

Participant 1, female, aged 61 years

The one participant who indicated that he would not want AI to be used in the shared decision-making process was the most technology-averse participant interviewed in this study. Throughout the interview, he made the following statements:

Well, these days everything runs by the computer. The people can’t live without it. But I’m not one of them. It’s worrying to me when you’ve got to listen to a computer. I’m not into technology. If we’ve got to keep living with technology, my days are up.

Participant 5, male, aged 70 years

This participant had no prior understanding of AI, and his aversion persisted after being provided with a working definition and clinical examples.

## Discussion

### Principal Findings

Thematic saturation was achieved with 19 interviews. The 20th interview confirmed that no new themes were emerging. The sample comprised 11 (N=20, 55%) female patients and 11 (N=20, 55%) patients with a postoperative complication substantial enough to require deviation from the routine postoperative course. The median age of the sample was 66 years. Three themes, each comprising 3 code categories, with each code category comprising 3 codes, captured the participants’ understanding of AI and their perceptions of its use in shared clinical decision-making. The theme *Expectations* captured participants’ views of themselves as individuals with the right to self-determination as they sought therapeutic solutions tailored to their circumstances, needs, and desires, including whether to use AI in the first place. The theme *Empowerment* highlighted the potential for AI to facilitate the development of realistic expectations and equip patients with personalized risk information to discuss with their surgeons to inform shared clinical decision-making. The theme *Partnership* captured the importance of a cooperative relationship between AI and the clinician to harness the clinician’s empathy and ability to interpret and understand human emotions where AI is lacking in this domain.

This is the first study to reveal the diverse conceptualizations and perceptions of patients who underwent knee replacement surgery regarding the use of AI for risk prediction in shared decision-making. The findings suggest that patients with varied perspectives and preferences can arrive at a shared functional understanding of AI, enabling them to consider its practical implications in shared clinical decision-making regarding their choice to consent to surgery. Although most study participants could see a role for AI in supporting clinical decision-making, there were mixed views on the importance of interpretability versus performance and preference for the clinician or patient to have the final say regarding whether to use AI. This is broadly consistent with prior research showing that patients are open to the use of AI, provided that it is not given full autonomy [42]. However, this study expanded upon this prior research by demonstrating differences in individual preference for being in control of the decision to use AI in shared decision-making or whether this control should be in the hands of the surgeon. The findings of this study suggest that AI could be engineered in such a way, and the information it generates used in such a way, to enable patients to be comfortable using it for risk prediction in shared clinical decision-making. It is important to reiterate that this is not a representative sample. Rather, the aim of this study was to conduct an in-depth exploration of a diverse range of perspectives.

### Comparison With Prior Work

The findings presented herein build upon prior qualitative work pertaining to decision-making in patients undergoing knee replacement surgery [2] by specifically exploring their views on AI for risk prediction in shared decision-making.

The *Expectations* theme captured the way participants viewed themselves as individuals seeking therapeutic solutions tailored to their circumstances and needs. Uniqueness neglect was a concern for some participants, whereby they emphasized the importance of personalized information and care [43,44]. Consistent with prior research [22], participants in this study expressed a desire for the surgeon to retain the power to make a decision that was contrary to the AI’s recommendation if the surgeon perceived something unique about the patient that the AI could not detect or did not give sufficient weight. Public trust in doctors has changed over time since the era of paternalistic medicine, and careful consideration is required regarding how AI might influence trust and shared decision-making [45]. This could prove to be crucial in what may become a new era of medical paternalism, where both the patient and the clinician are beholden to black box AI systems [46]. Prior research has also shown that patients may not be interested in AI performance gain above parity with clinicians’ performance if it necessitates sacrificing model explainability [47]. However, the patients interviewed in this study were evenly divided in terms of their willingness to compromise interpretability for an increase in predictive performance.

The suggestion raised in this study that patients could use the AI decision aid before their consultation with the surgeon raises the possibility that AI could equip them with more personalized information for discussion during the consultation [48]. This could relieve some of the pressure on the surgeon to do the busywork of entering information into the computer and free up time in the clinic for person-centered interaction [49].

The *Empowerment* theme highlighted the potential for AI to enable participants to develop realistic expectations and facilitate shared decision-making and personalized care [50]. AI decision aids can improve the efficiency, even if they do not outperform clinicians in terms of predictive accuracy. This has been demonstrated with an AI tool used in the follow-up of patients after orthopedic surgery [51]. AI could be integrated into patient follow-up systems, empowering patients to track their own postoperative recovery and report to their clinical team. The findings of this study align with prior work indicating that patients with osteoarthritis seem to be open to this type of digital intervention [52]. However, the concern raised in this study regarding data privacy breaches targeting AI systems has been raised previously [53,54], suggesting growing awareness and concern among patients. Furthermore, concerns regarding personal liability in cases where patients go against the recommendation of AI highlight the importance of good communication with their clinician. Clinicians need to be able to convey the information to the patient in a manner that they understand and that helps to properly inform their decision to undergo surgery [55], even if it is against the AI’s advice.

The *Partnership* theme speaks of the importance of symbiosis between AI and clinicians. Participants believed that AI could give the clinician useful information, but human interaction with the clinician was still crucial because AI lacks empathy [24,56]. Consistent with prior literature, participants were open to AI being used, but not if it was autonomous [22,40,57-60]. However, participants were evenly divided regarding their preference for a hypothetical trade-off between predictive performance and AI interpretability. This suggests that it cannot be assumed that all patients would prefer interpretability at the cost of predictive performance gains.

Many participants were open to the use of AI to enhance existing processes. This finding was consistent among those participants who would prefer the advice of AI to that of the clinician if the AI’s advice aligned more with their personal views. In such situations, participants still highly valued their autonomy, having the ability to choose the AI’s advice over that of the clinician [42]. Patients’ perspectives are critical in understanding how best to harness the potential of AI without blindly trusting the hype or properly considering its limitations [61].

### Implications of These Findings and Future Directions

Inspired by prior work [23], the findings of this study could be used in the process of developing a questionnaire to ascertain the views of patients undergoing knee replacement surgery on the acceptability of AI in shared clinical decision-making regarding risk prediction. Such a questionnaire could facilitate the inclusion of a large sample of patients that is more representative of the broader patient population that undergoes knee replacement surgery as well as being more culturally, socioeconomically, and linguistically diverse. This could present an opportunity to build upon the findings of population-based survey studies [17] and dig deeper into the novel findings of this study, including individual preferences concerning the trade-off between AI predictive performance and interpretability and individual preferences of patients regarding whether they should have the final say in the decision to use AI decision aids or whether this decision should be made by the surgeon. It could also encourage participation by patients who do not have time to participate in a qualitative interview but could feasibly complete a questionnaire. This could also facilitate the targeted dissemination of educational materials to improve the understanding of AI, thus informing their decisions to use this technology.

The objective of this study was not to assess the accuracy of patients’ understanding of AI. Rather, it was to explore what they understood AI to be and to delve deeper into their views on its use in shared clinical decision-making. Prior work has indicated that familiarity with AI is not associated with trust in AI [59]. However, future work could investigate the accuracy of the understanding of AI by patients undergoing knee replacement surgery, beyond familiarity with it, and how this influences their acceptance of its use in shared clinical decision-making. Some might be open to AI for risk prediction in shared clinical decision-making, provided it is explained to them comprehensively [62].

This study adds to the growing body of literature concerning patients’ perspectives on AI [63]. This is the first qualitative exploration of the views of patients undergoing knee replacement surgery regarding AI use in shared clinical decision-making. The use of qualitative semistructured interviews was built upon extensive survey studies [22] to gain a detailed understanding of the diverse perspectives of members of this patient population. The findings in this study were consistent with prior research [22] in that AI could be viewed as a useful second opinion to clinicians, but they differed in that AI was seen by the participants in this study as a support, rather than a threat, to shared decision-making, and participants generally were quite trusting of AI, assuming certain conditions were met. These conditions were that AI should not be used to deny treatment, it should be of proven high quality and reliability, and the human element of the clinician-patient interaction should not be degraded by the implementation of AI. Rather, AI should be used to do the number crunching and risk evaluation tasks such that there is more time available for human interaction between patients and clinicians [64]. The findings from this study may also be applicable to other orthopedic populations, such as patients undergoing hip replacement, and to patients undergoing knee replacement surgery with similar demographic characteristics in other parts of Australia and in other countries.

### Strengths and Limitations

A consumer advocate (author MG-H) was involved in the development and pilot testing of the interview guide to ensure that the questions were relevant and comprehensible. Their subsequent participation in regular discussions with the study coordinator (DJG) throughout the analysis, as well as their participation in group discussions pertaining to thematic analysis, enhanced the rigor of the analysis by ensuring that it was grounded in lived experience.

Participants with a broad range of demographic characteristics were recruited from a large and diverse referral base. The sample size was typical of qualitative studies, and saturation was achieved. The interview guide, coding framework, and participants’ quotes were presented to enable readers to understand how the interpretations of the data elucidated in this study were ascertained. Therefore, the views captured in this study may be applicable to other Australian settings serving similar patient populations, such as patients undergoing other elective surgeries, as well as international settings with similar health care systems.

However, the following limitations need to be considered. First, patients who consented to participate may differ from those who declined in terms of their affinity to AI and their capacity to understand the conceptual basis of this technology and consider the implications of its implementation for risk prediction in shared decision-making. The impact of this limitation was reduced by checking the participants’ understanding of AI before being provided with the same working definition as all other study participants. It was also made clear to the participants in the study recruitment material and verbal consent script that they could ask any questions for clarifications and that there were no prerequisites in terms of knowledge of, or affinity to, AI. Second, due to the restrictions imposed in light of the COVID-19 pandemic, interviews were conducted via telephone. This meant that body language could not form a part of the interview, potentially reducing the effectiveness of communication and explanation throughout the interview. In order to reduce the impact of this limitation, a family member or other support person was allowed to be present for the interview to check the understanding and assist with communication. Third, due to the COVID-19 pandemic, elective surgeries in Victoria, Australia, were canceled, meaning that patients with recent surgeries were not available for recruitment. As such, some patients had their surgery a few years prior, which increased the likelihood of recall bias regarding their experience. However, to mitigate the impact of this limitation, recruitment was targeted to patients who had their surgery as close to the study period as possible.

### Future Directions

Future work could address some of the limitations of this study. For example, people undergoing knee replacement surgery could be interviewed at a common time point shortly after surgery to reduce recall bias. Interviews could also take place in person, facilitating the use of visual aids to assist participants in understanding definitions and hypothetical scenarios.

The development of the infographic depicted in Figure 3 was an extension of DJG and MG-H’s collaborative partnership throughout DJG’s PhD project, which involved developing an ML risk prediction tool [65]. This educational flyer requires pilot testing and further refinement before it can be deployed in the clinical environment. Future qualitative research could explore the educational effect of the flyer along with illustrative scenarios for patients undergoing knee replacement surgery regarding the use of AI for risk prediction in shared decision-making as well as the role that clinicians could play in enhancing patient understanding of AI.

### Conclusions

Patients who underwent knee replacement surgery are likely to be faced with more AI decision aids in the shared clinical decision-making process. These patients may have varied levels of familiarity with AI and diverse conceptualizations of its definitions and capabilities. Despite this heterogeneity of familiarity and technical understanding, participants in this study were mostly open to the use of this technology in shared clinical decision-making, provided that their personal autonomy, needs, and values were respected. Educating patients on what AI is, what it can offer, and what its limitations are could help inform their decision regarding whether they wish to have such tools used in their shared decision-making process around consenting to surgery. Clinicians may play a key role in educating patients and finding a place for AI in the clinical setting as the uptake of this technology in health care continues to grow.

## Appendix

Multimedia Appendix 1 COREQ (Consolidated Criteria for Reporting Qualitative Research) checklist.

Multimedia Appendix 2 Qualitative interview guide.

Multimedia Appendix 3 Code descriptions.

Multimedia Appendix 4 Code category descriptions.

Multimedia Appendix 5 Demographic representation in each theme.

Multimedia Appendix 6 Responses to questions that required a binary response.

## Abbreviations

AI: artificial intelligence
COREQ: Consolidated Criteria for Reporting Qualitative Research
MD: doctor of medicine
ML: machine learning
PhD: doctor of philosophy

## Data Availability

The data sets generated and analyzed during this study are not publicly available for confidentiality reasons but may be available from the corresponding author on reasonable request; this would likely involve a proposal for a collaborative project requiring separate ethics approval to use deidentified qualitative interview transcripts.
