# Supplementary material for: Patients’ Views on AI for Risk Prediction in Shared Decision-Making for Knee Replacement Surgery: Qualitative Interview Study
Source: J Med Internet Res. 2023 Sep 18;25:e43632. doi: 10.2196/43632 (PMC10546266; doi:10.2196/43632)
Supplement: Multimedia Appendix 2 [file jmir_v25i1e43632_app2.pdf]

Qualitative interview guide

RESEARCH QUESTION = What do total knee arthroplasty patients understand about artificial intelligence, and what are their perceptions on the use of artificial intelligence in the shared clinical decision-making process?

1. Opening statement: REMINDER OF WHAT THE STUDY IS ABOUT AND THAT THE PATIENT HAS CONSENTED TO PARTICIPATE - GET THE PATIENT TO GIVE VERBAL CONFIRMATION – THEN PROCEED (that way a reminder of consent is on record) followed by: "there is no right or wrong answer to any question. Rather, I am interested in **your** understanding and opinions. This is a confusing, highly technical topic. Many people who claim to be experts still mix up the terminology."

2. We will start by exploring your personal perspective, as a patient:
    a. Where do/did you get your information regarding TKA surgery as a treatment option for you?
        i. What about GP, friends and family, other patients, surgeons (multiple opinions)?

    b. How did you go about making this decision?
        i. Discuss with family and friends? Research on the internet?

    c. What is your process for weighing the pros and cons, benefits and risks, etc.?

    d. How do you deal with conflicting information? E.g. different opinions from friends and family, physiotherapists, online information.

    e. What input DID your **surgeon** have in deciding whether an operation is a good option or not?

    f. What sort of information do you think the surgeon takes into account?
        i. What were some of the risks they talked to you about?

    g. What input DID you have, as a **patient**, in deciding whether an operation is a good option or not?

h. What information did the surgeon discuss with you?

i. What sort of questions did you ask the surgeon?

3. So, one of the things we are interested in is the role that computers and artificial intelligence might play in supporting patients and surgeons to make decisions about surgery. I am going to change gears now and ask you a little about your thoughts and attitudes towards technology more broadly. Again, there is no right or wrong answer:
   a. Tell me about your use of technology (computers, phones, appliances, etc.) in everyday life.

   b. What does the term ‘Artificial Intelligence’, or ‘AI’ mean to you?

   c. Functional definition of AI = A computer system that takes information and LEARNS FROM IT to make decisions based on rules it comes up with. A human doesn’t tell it exactly how to arrive at its decision, therefore it is often referred to as a ‘black box’ because we have little to no idea exactly how it makes that decision. E.g. Deep Blue, the algorithm that beat the world chess champion in 1997.

      i. Does that make sense?

   d. So the way these things are built, for example in surgery, is you have, say, 5000 patients and you have their information. Let’s say 500 of them have a complication after surgery. The AI LEARNS how to tell the difference between the 500 and the rest of the 4500. When the AI is used on a new patient, the perfect AI always gets it right and never misses a patient who has a complication. In real life, they’re not perfect; sometimes AI will miss patients and sometimes it will say a patient is going to get a complication and it turns out they don’t. A bad AI tool is no better than flipping a coin, i.e. chance. In the real world, AI tools are somewhere in between these.

      i. Does that make sense?
      ii. Check that the patient understands ‘risk’ – can think of this as likelihood or ‘chance’ of something happening

   e. Based on information about you as a patient, what would you like to know about before having surgery? Imagine the AI can find out anything, including

good and bad outcomes. (FOR EXAMPLE any complications you would want to avoid, any positive outcomes you would want to achieve)

i. IF PATIENT HAD A COMPLICATION, SUGGEST THEY THINK ABOUT THAT EXAMPLE (**reiterate to them that none of this information will be passed on to their surgeon**)

f. (IF THE PARTICIPANT CAN THINK OF AN EXAMPLE, USE THEIR EXAMPLE. IF NOT, USE THE FOLLOWING) Imagine the following scenario: There is an AI tool that predicts the risk of you, the patient, being readmitted to the hospital within 30 days after TKA surgery (OR METAL-ON-METAL HIP REVISION, i.e. make it relevant to each participant if possible – otherwise just use 30d readmission). Let's say our tool has pretty good accuracy not perfect, but definitely better than a coin toss.

i. Does that make sense?

ii. How do you feel about such a tool being used in your shared decision-making process with your surgeon? (CONTINUE TO USE THEIR EXAMPLE (or use readmission if necessary))

iii. What do you think are the advantages/pros/benefits of AI in shared clinical decision-making? (Lay examples include restaurant/song recommender systems, GPS, etc. Already given medical example(s))

iv. What do you think are the disadvantages/cons/risks of AI in shared clinical decision-making? (Lay examples include restaurant/song recommender systems, GPS, etc. Already given medical example(s))

v. What if you had made up your mind that you really wanted surgery, but the AI tells you that you are at high risk of (WHATEVER EXAMPLE YOU DECIDE UPON WITH THE PATIENT, OR READMISSION IF THEY'RE STUCK). How would this affect your decision to proceed to surgery?

vi. If you were told that 80% of all TKR patients do well from surgery, what would you think? What about if it was 60%? (THIS QUESTION WAS ADDED LATER IN THE STUDY AND WAS NOT INCLUDED IN THE MAIN ANALYSIS)

vii. If you were told that 70% of TKA patients LIKE YOU do well from surgery, what would you think then? What about if it was 50%?"

viii. What about if you were told that 30% of people LIKE YOU will experience an infection or other complication from surgery? What if it was 50%?

ix. Say there is such a tool available. Rather than the surgeon automatically using it for every patient, they give you a choice. Would you want your information used with the AI tool in your shared decision-making?
    1. Why/why not?
    2. What if the surgeon does not want to use it, but the patient does. Who has the final say? Why?

x. What should the surgeon do with the information from the tool?
    1. Under what circumstances should the surgeon be able to go against the recommendation of the tool? – (Back-up questions if participant needs more detail: for example, offer surgery even if the tool says you are high-risk of that particular outcome? What about if we know that the AI is better at predicting the outcome than the surgeon?)

xi. How important is it that the surgeon has been compared to the tool on the same task?

xii. Which of the following AI tools would you prefer?
    1. AI 1 = Reasonably accurate (better than a coin toss), and the people who made it know how it thinks, i.e. not a black box
    2. AI 2 = Black box, i.e. no one knows how it thinks, not even the people who made it, but it is more accurate, i.e. much better than a coin toss

xiii. If you chose AI 1, why do you think knowing how the tool thinks is more important than accuracy?

xiv. If you chose AI 2, why do you believe accuracy is more important than knowing how the AI thinks?

4. Back-up examples, if participants require/request more context:
    a. Example of ‘big data’ (not AI, but data from a lot of people) = AOANJRR was used to detect the ‘metal on metal’ problem leading to high THA revision rates: https://www.ncbi.nlm.nih.gov/pmc/articles/PMC5435639/

   b. Who is collecting the information? For what purpose? = sometimes specifically for research (e.g. SMART registry); sometimes ‘administrative’ data routinely collected as you go through the healthcare system
   c. From participant 4: “Whereas when you're talking about the infection, I mean, if it's got to database full of, you know, a million knee reconstructions, it's got all that information in its hands. A doctor may have done a thousand, but the doctor has to think back and go through it whereas a computer wouldn't have to worry. It just knows.”
5. Back-up questions (if there is time remaining):
   a. Do you think AI will be more accurate, equally accurate, or less accurate than clinicians for predicting readmission? Why?
   b. Why?/Could you expand on that?/Anything else to add?
   c. The documentary ‘Coded Bias’ uncovered racial discrimination in face recognition software because it did not recognised dark-skinned faces. The AI tool was not built with enough examples of dark-skinned faces to recognise them. Are you concerned about AI tools in shared clinical decision-making making similar errors? Imagine you are one of the people who is very different from the patients whose information was used to build the tool. How does that make you feel about the use of such a tool in shared clinical decision-making?
   d. Why?/Could you expand on that?/Anything else to add?
