# Supplementary material for: Patients’ Views on AI for Risk Prediction in Shared Decision-Making for Knee Replacement Surgery: Qualitative Interview Study
Source: J Med Internet Res. 2023 Sep 18;25:e43632. doi: 10.2196/43632 (PMC10546266; doi:10.2196/43632)
Supplement: Multimedia Appendix 3 [file jmir_v25i1e43632_app3.pdf]

Table A1 – Code descriptions

Code	Description
Decision-making process	The patient's process for making decisions, particularly regarding surgery. This includes, but is not limited to, the decision to have surgery in the first place.
AI in shared decision-making	The patient's understanding of, and/or views on, the role of AI in shared decision-making with the clinical team. In particular, the process by which AI obtains and uses information and the process by which it is used or consulted – depending on whether the patient conceptualises AI as an information-giving tool or as an entity on its own.
Health system processes	The mechanisms in place to which patients are subject and which they must navigate to receive knee replacement. This includes the way in which information is collected and used, as well as the way in which the interaction between patient and surgeon is allowed to, expected to, and tends to take place. Also extends to interactions with GP and other health system/clinical staff.
Weighing sources of information	The patient's way of judging the relevance and importance of the various sources of information presented to them, such that they come to a decision regarding whether to proceed with surgery.
Shared decision-making	Utilising the clinician's judgement and communication, in conjunction with the patient's personal judgement and autonomy, to jointly decide upon a course of management. This could include other parties, e.g. a family member. Could also, obviously, include AI, especially if the patient considers AI to be a thinking entity of sorts (but that is a separate code of its own – AI in shared decision-making)
Resolve	The firmness of the patient's decision to have surgery. Patients may not necessarily be particularly decisive, but by the time they see the surgeon regarding knee replacement they often have their mind made up and perceive a lack of viable alternatives.
Outlook	Outlook = The patient's attitude towards the clinical partnership with their surgeon, and towards healthcare more broadly, as it pertains to surgery as a potential treatment option to address their needs. Includes optimism or pessimism regarding potential outcomes of surgery compared to the natural history of their osteoarthritis.
Modifying risk	The perceived ability, or lack thereof, to have their likely prognosis altered in a positive or negative way through their own actions, or actions taken on their behalf based on information pertaining to them.
Personal vs general prognosis	The patient's understanding and perception of the way in which their individual risk profile can differ from that of the knee replacement population average, and how this information can inform the decision-making process.
Patient's responsibility	The role the patient understands they are expected to play in both the shared decision-making process regarding whether to have surgery, and

	their subsequent pre- and post-operative course in order to maximise their likelihood of a good outcome.
Clinician's responsibility	The role the patient understands the clinician is expected to play in the shared decision-making process, including communicating information about risks as well as risk:benefit ratio and realistic expectations of what sort of benefits surgery can provide as well as the magnitude of those benefits, while respecting patient autonomy, regarding appropriateness of knee replacement as a treatment option.
AI as responsible entity	Patient understands/perceives AI to be not just an information source or information processing unit, but a mind of its own, in a sense, which is capable of making decisions and/or offering advice as if it were a thinking third party.
Patient self-efficacy	The patient's perception of their own ability to make sure their voice is heard and their wishes are respected.
Role of surgeon in shared decision-making	Patient's familiarity with the role the surgeon plays in the patient's knee replacement journey, including who gets the final say regarding knee replacement as a treatment option and what the surgeon is supposed to discuss with the patient.
Awareness of risks	General awareness of risks of knee replacement, risks of using AI as decision aid, etc., as well as awareness of their specific risks as an individual based on their own characteristics which differ from the population average. This can arise through discussion with GP or surgeon, or through close contacts with experience of knee replacement, or through independent research and/or experience.
Understanding of AI	The patient's own understanding of AI, before and after a working definition is given during the interview. Correctness of their understanding is not the important aspect of this code. The important thing o capture is how he patient conceptualises it.
Understanding of risk	Patient's understanding of risk as a concept, and of risks specifically associated with knee replacement. Again, the correctness of their understanding is not the important aspect here. It is the patient's perspective that matters.
Understanding of appropriateness of KNEE REPLACEMENT	Combined understanding of indications for knee replacement and likely success of the procedure for the patient as an individual, relative to alternative potential management options which may or may not have been explored.
Clinician's influence	The patient's impression of the clinician's ability to sway the patient's decision-making, both overtly/explicitly and in more subtle, cryptic, unintentional ways, such as through the power imbalance in the clinical partnership. The clinician may not be aware of this influence; indeed, in many cases they are probably unaware of it.
AI as decision aid	The patient's understanding of the amount of power AI has to influence the decision-making process. This is related to the patient's

	understanding of AI as a piece of information, an information-generating unit, or an independent thinking entity.
Trust in clinicians	Patient trusts that the surgeon would not offer surgery if it were inappropriate. There is a sense in which risk has been factored in, broadly, and the clinician has deemed the risk:benefit calculation to be in the patient's favour.
Trust in AI	Patient's level of trust in AI generally, and/or specifically in the context of shared decision-making regarding knee replacement as a treatment option. There may be different levels of trust for different types of AI and/or different applications of it.
Trust in self	Patient's self-assuredness and degree of confidence in their own ability to make the best treatment decision. This includes their ability to appraise information, communicate the severity of their condition and its effect on their life, and seek relevant information if they feel there is something they need to know but about which they have not yet been informed.
Familiarity with AI	Whether, and to what extent, the patient is aware of AI. This also incorporates the way in which the patient gained this awareness, e.g. through media or job experience. This is separate to understanding of AI in that the patient can be familiar with AI/aware of it, but completely misunderstand what it is or claim to not have any understanding of what it is.
Concerns	Very broad concept capturing the patient's personal concerns regarding knee replacement, decision-making, AI, etc. This is tied to the patient's values in that what concerns them will not necessarily concern others and vice versa.
Beliefs	Patient's beliefs regarding how shared decision-making should take place, how AI should play into the process, if at all, and other factors such as how the surgeon should carry out their role. Patients also often have quite firm views on their own autonomy. This code also applies to beliefs about AI, the healthcare system, the skill of the surgeon, etc. which play an important role in the decision-making process when there are unknowns and therefore trust is required on the basis of these beliefs.
Rights	Rights of the patient in the shared decision-making process, and rights of the surgeon in this process. Could also entail rights of AI if patients see it as an independent entity, but I don't believe this is applicable to my dataset. This stems from the patient's beliefs and values in that they might not know their legal rights but have a sense of what they ought to be.
