# Supplementary material for: Patients’ Views on AI for Risk Prediction in Shared Decision-Making for Knee Replacement Surgery: Qualitative Interview Study
Source: J Med Internet Res. 2023 Sep 18;25:e43632. doi: 10.2196/43632 (PMC10546266; doi:10.2196/43632)
Supplement: Multimedia Appendix 4 [file jmir_v25i1e43632_app4.pdf]

Table A2 – Code category descriptions

Category	Description
Process	Refers to the way in which decisions are made regarding knee replacement surgery. This includes the processes of the healthcare system within which all decision-making must take place and which influences all aspects of the decisions patients make, even though patients may not be cognizant to these processes and their intricacies. It also includes the patient's decision-making process and the way in which artificial intelligence might be used here.
Judgement	Not in a moral or condemnatory sense. Rather, judgement refers to the patient's role as the ultimate decision-maker regarding their surgery. Various sources of information, involving various different parties, are at play. The patient is on a journey which leads them to the point of making the decision once they are satisfied with the information given to them and the way in which they have made up their mind.
Prognosis	The patient's understanding of how knee replacement could benefit them in relation to the potential harms to which it could expose them. Patients may or may not be aware of their outlook regarding knee replacement surgery, and as such their optimism or pessimism can be revealed explicitly or implicitly. Prognosis is also understood to be a dynamic thing which can be influence in either a positive or negative way through various actions. The patient appreciates their own prognosis, as an individual with unique characteristics, in relation to the broader knee replacement population.
Responsibility	The patient understands they have responsibilities both to make the final decision regarding whether to proceed with surgery, and what they ought to do to increase their likelihood of a good outcome. This is in relation to the responsibility of the clinicians with whom the patient interacts along their journey, but also with the artificial intelligence. The latter can be seen as a tool available to assist the former, or as both a tool and an independent conscious entity of its own such that the AI has responsibility also.
Power	There is a complex interaction occurring between the patient and clinician. There is an obvious power imbalance, however the paradigm of patient-centred care is vastly different from the overtly paternalistic system of yesteryear. The patient ultimately has the final say in whether they proceed with surgery, but having all of the pertinent information available to make such a decision in an informed manner is not straightforward. The patient may even want to relinquish some of their power, as it were, to the surgeon if they do not personally feel capable of making what they would deem the best decision. Introduce AI into the interaction and there is the possibility of empowering both parties, but the output from AI should ultimately not be used to dictate terms. Rather, it should be used primarily to empower the patient to make a better-informed decision regarding surgery.

Trust	Although family members and friends are somewhat involved, there are three main parties in which trust must be present during the patient's journey to knee replacement: clinicians, AI, and self. Surgeon and GP usually comprise the clinician group and trust in self is fairly self-explanatory, but trust in AI is a bit more complex. The patient may understand the AI to be an independent, thinking entity therefore trusting it is akin to trusting human decision-makers. They may instead understand it to be a computer tool built by humans which does not necessarily think for itself. In either case, trust in the people who built it and trust in the tool itself are key determinants in how much trust the patient places in AI, and whether they even choose to use it at all.
Awareness	Distinct from understanding, awareness refers to the patient's baseline level of familiarity with various concepts introduced throughout the interview. They may offer this spontaneously in the course of the interview, or they may need to be prompted. In some cases, this is essentially a step before understanding; i.e. a patient must be aware of AI on some level if they understand it. In other cases, it refers to a broader awareness of the implications of, and context surrounding, the topics discussed throughout the interview.
Understanding	Fairly straightforward category capturing the patient's understanding of AI, directly in line with the research question, but also their understanding of risk and of the appropriateness of knee replacement. The latter two codes were included because the ultimate decision the patient makes is whether to have knee replacement surgery. The risk/benefit consideration seems to form the core of this decision. As such, the patient's understanding of what risk is and what it means in relation to their decision-making is critical to explore. The appropriateness of TKA, according to the patient, is a function of the patient's understanding of risk and what it means for them in relation to the perceived benefit of surgery. This includes understanding the nature of risk as a concept, as well as the specific nature of the various risks and their severity.
Values	The decision to have knee replacement surgery is quite substantial and, as such, often brings to the fore the patient's values and beliefs. These can be deep-seated and the patient may not be directly aware of their nor be able to articulate them clearly. As various topics are discussed throughout the interview, patients reveal their values and beliefs. It is at the intersection between these values and beliefs, and the situations and concepts discussed in the interview, that the patient's concerns emerge regarding AI technology, surgery, decision-making, etc.
