# Supplementary material for: Patients’ Views on AI for Risk Prediction in Shared Decision-Making for Knee Replacement Surgery: Qualitative Interview Study
Source: J Med Internet Res. 2023 Sep 18;25:e43632. doi: 10.2196/43632 (PMC10546266; doi:10.2196/43632)
Supplement: Multimedia Appendix 5 [file jmir_v25i1e43632_app5.pdf]

Table A3 – Demographic representation in each theme

Theme	Total number of substantive quotes = n	Age groups = n (% proportion of total)	Gender = n (% proportion of total
Expectations	747	<60 = 187 (25.0%)<br>60-70 = 213 (28.5%)<br>>70 = 347 (46.5%)	M = 329 (44.0%)<br>F = 418 (56.0%)
Empowerment	676	<60 = 183 (27.07%)<br>60-70 = 188 (27.81%)<br>>70 = 305 (45.12%)	M = 293 (43.3%)<br>F = 383 (56.7%)
Partnership	475	<60 = 108 (22.7%)<br>60-70 = 150 (31.6%)<br>>70 = 217 (45.7%)	M = 222 (46.7%)<br>F = 253 (53.3%)
