# Supplementary material for: Patients’ Views on AI for Risk Prediction in Shared Decision-Making for Knee Replacement Surgery: Qualitative Interview Study
Source: J Med Internet Res. 2023 Sep 18;25:e43632. doi: 10.2196/43632 (PMC10546266; doi:10.2196/43632)
Supplement: Multimedia Appendix 6 [file jmir_v25i1e43632_app6.pdf]

Table A4 - Responses to questions which required a binary response

**Preference**	**Response = n (% proportion of total)**
Had a baseline understanding of AI before being given definition	Yes = 11 (55%)<br>No = 9 (45%)
Would use AI tool in shared clinical decision-making if given the option	Yes = 19 (95%)<br>No = 1 (5%)
